# MUC1 gene polymorphisms are associated with serum KL-6 levels and pulmonary dysfunction in pulmonary alveolar proteinosis

**DOI:** 10.1186/s13023-016-0430-2

**Published:** 2016-04-23

**Authors:** Francesco Bonella, Xiaoping Long, Shinichiro Ohshimo, Yasushi Horimasu, Matthias Griese, Josune Guzman, Nobuoki Kohno, Ulrich Costabel

**Affiliations:** Interstitial and Rare Lung Disease Unit, Ruhrlandklinik, University of Duisburg-Essen, 45239 Essen, Germany; Department of Respiratory Medicine, The First Affiliated Hospital of University of South China, Hengyang, Hunan P. R China; Department of Emergency and Critical Care Medicine, Graduate School of Biomedical Sciences, Hiroshima University, Hiroshima, Japan; Department of Molecular and Internal Medicine, Graduate School of Biomedical Sciences, Hiroshima University, Hiroshima, Japan; Dr. von Haunersches Kinderspital, University of Munich, Munich, Germany; General and Experimental Pathology, Ruhr University, Bochum, Germany

**Keywords:** Pulmonary alveolar proteinosis, KL-6, MUC1 polymorphisms, Disease outcome

## Abstract

**Background:**

KL-6, a human MUC1 mucin, is a sensitive biomarker for interstitial lung diseases including pulmonary alveolar proteinosis (PAP). A correlation between MUC1 gene single nucleotide polymorphism (SNP) rs4072037 genotype and serum KL-6 levels has been reported. This study was aimed at investigating the correlation between MUC1 SNP genotype, severity of disease and disease outcome in PAP.

**Methods:**

Twenty four patients with PAP and 30 healthy volunteers were studied. MUC1 rs4072037 was detected by using a real-time polymerase chain reaction (RT-PCR). Genotyping was performed by pyrosequencing. KL-6 levels were measured in serum by Nanopia KL-6 assay (SEKISUI Diagnostics).

**Results:**

The frequency of MUC1 rs4072037 alleles was significantly different between PAP patients and healthy volunteers (PAP, A/A 46 %, A/G 54 %, G/G 0 %; healthy controls, A/A 30 %, A/G 40 %, G/G 30 %; *p* = 0.013). Serum KL-6 levels were significantly higher in PAP patients than in controls (*p* < 0.0001), and significantly higher in PAP patients with A/A genotype than in those with A/G genotype (*p* = 0.007). Patients with A/A genotype had higher alveolar-arterial oxygen difference (A-aDO_2_) and lower DLco compared to those with A/G genotype (*p* = 0.027 and *p* = 0.012, respectively). Multivariate analysis, Kaplan-Meier analysis and C statistics showed that the rs4072037 A/A genotype was associated with higher rate of disease progression (HR: 5.557, *p* = 0.014).

**Conclusions:**

MUC1 rs4072037 A/A genotype is associated with more severe pulmonary dysfunction and a higher rate of disease progression in PAP patients.

## Background

Pulmonary alveolar proteinosis (PAP), first described in 1958 [1], is a rare syndrome (1–3 cases per million) characterized by the intra-alveolar accumulation of surfactant lipoproteins [2]. The most common form is autoimmune and is associated with the presence of autoantibodies against GM-CSF [3–7].

KL-6 is a mucin-like glycoprotein belonging to the MUC1 family [8]. KL-6 is moderately expressed in type II pneumocytes and respiratory bronchiolar epithelial cells and only weakly expressed in basal cells of the terminal bronchiolar epithelium of normal lung tissues [8]. Regenerating alveolar type II pneumocytes are the primary cellular source of KL-6 in the affected lungs of patients with interstitial lung diseases (ILD) [9–12]. Serum KL-6 is a sensitive biomarker for various ILD, such as idiopathic pulmonary fibrosis, radiation pneumonitis, drug-induced pneumonitis, hypersensitivity pneumonitis, CTD-associated ILD, pulmonary sarcoidosis, and cystic fibrosis [9, 13–16]. KL-6 levels have been found to be elevated in serum and bronchoalveolar lavage (BAL) fluid of PAP patients at a concentration in BAL 3–5 fold higher than in serum [12, 17].

It has been reported that the rs4072037 single nucleotide polymorphism (SNP) in the exon 2 of the MUC1 gene is associated with inter-individual variability of serum KL-6 levels [18, 19]. In addition, different distribution of the SNP genotypes between Caucasian and Japanese subjects has been observed [20].

The clinical utility of serum KL-6 in PAP has been only partially investigated [12, 17, 21–25]. Recently our group showed that serum KL-6 levels are a strong predictor of disease progression and of the necessity of treatment with whole lung lavage (WLL) in PAP patients [21]. However, the distribution of MUC1 SNP genotype and how this affects serum KL-6 levels is unknown in PAP patients.

The aim of this study was to investigate the correlations between MUC1 SNP genotype distribution, serum KL-6 levels, severity of disease and disease outcome in PAP patients. Some of the results of this study have been previously reported in the form of an abstract [26].

## Methods

### Disposition of the patients

We retrospectively studied 24 Caucasian patients with autoimmune PAP followed in our institution between 2007 and 2014. The diagnosis of PAP was based on characteristic BAL, high resolution computed tomography (HRCT), and/or histopathologic findings [5, 27]. GM-CSF autoantibodies were detected in all patients (Table 1). As a comparison group, 30 healthy controls were also included. The study was approved by the local IRB (approval number 10–4397). Written informed consent was obtained from both patients and healthy controls.

**Table 1:** Demographics and baseline characteristics of the studied subjects

Variables	PAP	Controls	*p* value
	*n* = 24	*n* = 30	
Demographics			
Age, years	46 ± 2	41 ± 2	n.s.
Gender, male/female, n	14/10	11/19	n.s.^b^
Smoking history, Current/Non, n	9/15	4/26	0.06^b^
BMI (kg/m^2^)	26 ± 1	24 ± 1	n.s.
Pulmonary function			
FVC (% pred)	80 ± 4	97 ± 7	<0.0001
FEV_1_ (% pred)	74 ± 3	93 ± 2	<0.0001
PaO_2_ (mmHg)	69 ± 3	84 ± 1	0.005
TLC (% pred)	81 ± 3	94 ± 3	0.025
SaO_2_ (%)	94 ± 1	95 ± 1	n.s.
A-aDO_2_ (mmHg)	40 ± 3	8 ± 1	<0.0001
DLco (% pred)	50 ± 21	86 ± 10	0.002
Biomarkers			
GM-CSF autoantibody (μg/mL)^a^	52 ± 6	4 ± 1	<0.0001
KL-6 (U/mL)^a^	5004 ± 983	283 ± 19	0.0001
LDH (IU/L)^a^	312 ± 24	193 ± 6	<0.0001

Unless otherwise indicated, values are expressed as mean ± SE.

*n.s.* not significant

^a^The cut-off of normality for each biomarker is reported in the methods

^b^Fischer’s exact test, all other comparisons with student’s *t*-test

### Definition of disease progression

Disease progression was defined as the necessity of treatment with WLL during follow-up. The indication for WLL was given on the basis of deterioration of self-reported symptoms (worsening of dyspnea, cough, chest pain and weight loss) and/or lung function (decrease in FVC or DLco >10 % pred or increase in the A-aDO_2_ > 10 mmHg), and/or chest imaging (increase of the previous findings or appearance of new infiltrates characteristic of PAP) since the last follow-up visit [21].

### KL-6, GM-CSF autoantibody and other laboratory assays

Serum samples were obtained by venipuncture at time of first evaluation and were stored at −80 °C until analysis. Serum KL-6 was measured by NANOPIA® KL-6 assay (SEKISUI Diagnostics, UK; upper limit of normal <458 U/mL as determined in 142 Caucasian healthy subjects). GM-CSF autoantibody (Abs) concentration was measured according to Kitamura, T., et al. [28]. Recombinant GM-CSF (Sargramostim, Genzyme, Cambridge, USA) was used to coat plates, as standard we used monoclonal human-anti-human GM-CSF antibody (BI01049904) provided by Boehringer Ingelheim, Germany. The detection limit of this assay is 0.2 μg/mL. GM-CSF Abs values <3 μg/mL are considered normal, 3–7 μg/mL intermediate, and >7 compatible with autoimmune PAP, according to Inoue et al. 2008 [4]. LDH was routinely measured in serum (normal value for LDH in our laboratory < 225 IU/L).

### DNA preparation and genotype analyses of MUC1 rs4072037

Genomic DNA was extracted from peripheral blood leukocytes by using silica-membrane- based nucleic acid purification Kit (Qiagen DNA Mini Kit, Qiagen, USA), and stored at −80 °C before use. As previously described [29], the rs4072037 genotype was determined using a real-time polymerase chain reaction (RT-PCR) method. We used a commercially available SNP genotyping assay (TaqMan SNP Genotyping Assay C 27532642–10; Life Technologies Corp. Carlsbad, California, USA) and the Applied Biosystems 7500 Fast RT-PCR System (Life Technologies Corp. Carlsbad, California, USA).

### Pulmonary function tests

Measurements included forced vital capacity (FVC), forced expiratory volume in one second (FEV_1_), total lung capacity (TLC), diffusing capacity of the lung for carbon monoxide (DLco), partial pressure of oxygen in arterial blood (PaO_2_), and alveolar-arterial oxygen gradient (A-aDO_2_). They were performed at time of the blood sample collection. Values were expressed as percentages of predicted normal values [30].

### Statistics

Continuous variables were evaluated for a normal distribution with the Kolmogorov-Smirnov test. Parametric data are presented as mean ± standard error of mean (SEM). Categorical variables are presented as either a percentage of the total or numerically, as appropriate. Comparison between two groups was done with Student’s *t*-test or Wilcoxon’s rank test for continuous variables, Chi-squared or Fischer’s exact test for categorical variables. Spearman’s or Pearson’s correlation coefficient was obtained for correlations. Pearson’s goodness-of-fit Chi-square test and Fisher’s exact test were used to test for deviation from Hardy-Weinberg equilibrium. Univariate and multivariate Cox proportional hazard regression model was conducted to study the independent effect of age, gender, smoking history, ethnicity, MUC SNP genotypes, and pulmonary function tests on the disease course. The Kaplan-Meier method with log-rank test was used to analyze whether MUC SNP genotypes were associated with the disease outcome. The predictive value for disease progression of each considered variable was evaluated by Harrell's C statistic. P values of < 0.05 were considered statistically significant. All statistical analyses were performed using SPSS 17.0 (SPSS Inc., Chicago, IL, USA).

## Results

### Demographics and patients’ outcome

Demographics and baseline characteristics of all studied subjects are shown in Table 1. The mean follow-up time of PAP patients was 18 ± 2 months (Range 1–36) from baseline blood sampling. All patients experiencing disease progression (*n* = 12) were treated with whole lung lavage (WLL), three of them received multiple WLL (>2) during follow up. At baseline, 21 patients had already received at least one WLL before blood sampling. Of them, 5 patients were in remission at time of blood sampling.

### Serum levels of KL-6

**Fig. 1:**
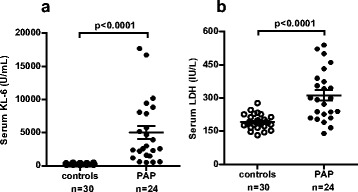
Comparison of serum levels of KL-6 (**a**) and LDH (**b**) in 30 healthy controls and in 24 PAP patients

The distribution of serum KL-6 levels was normal (*Z* = 0.727, *p* = 0.67). Serum KL-6 levels were significantly higher in PAP patients than in healthy controls (mean ± SEM: 5004 ± 983 vs. 283 ± 19 U/mL, *p* < 0.0001), and serum LDH levels were also higher in PAP patients than in healthy controls (312 ± 24 vs. 193 ± 6 IU/L, *p* < 0.0001) (Fig. 1).

### MUC1 SNP genotypes and biomarkers levels distribution

**Fig. 2:**
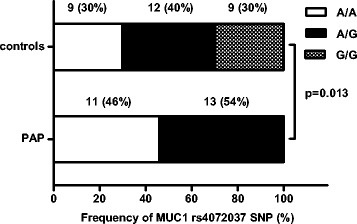
Frequency of MUC1 rs4072037 SNP genotypes in the studied subjects

MUC1 rs4072037 allele A and G were in Hardy-Weinberg equilibrium in the studied cohort (A =61 %, G = 39 %, *p* = 0.8). The frequency of MUC1 rs4072037 SNP genotype was significantly different between PAP patients and healthy controls (PAP: A/A 46 %, A/G 54 % and G/G 0 %; healthy controls: A/A 30, A/G 40 and G/G 30 %, *p* = 0.013) (Fig. 2).

**Fig. 3:**
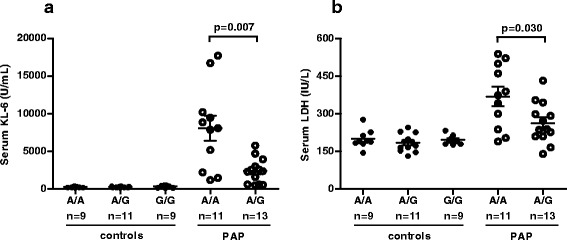
Correlation of MUC1 SNP genotype with serum KL-6 levels (**a**) and serum LDH levels (**b**) in 30 healthy controls and in 24 PAP patients

Serum KL-6 levels were significantly higher in PAP patients with A/A genotype than in those with A/G genotype (8084 ± 1673 vs. 2397 ± 462 U/mL, *p* = 0.007), whereas such genotype-related differences in KL-6 levels were not seen in healthy controls (A/A: 251 ± 25, A/G: 267 ± 20, G/G: 337 ± 40 U/mL, p > 0.05 for all comparisons) (Fig. 3a).

Serum LDH levels were also significantly higher in PAP patients with A/A genotype than in those with A/G genotype (369 ± 38 vs. 263 ± 22 IU/L, *p* = 0.030), but such differences were not seen in healthy controls (A/A: 200 ± 12, A/G: 185 ± 10, G/G: 196 ± 6 IU/L) (Fig. 3b).

### MUC1 SNP genotype and pulmonary dysfunction

**Fig. 4:**
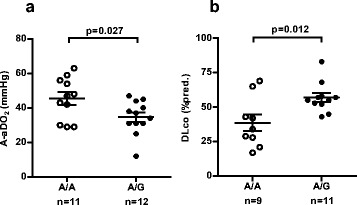
Correlation of MUC1 SNP genotype with A-aDO_2_ (**a**) and DLco (**b**) in PAP patients

Patients with A/A genotype had a higher A-aDO_2_ gradient and a lower DLco compared to those with A/G genotype (45 ± 4 vs. 35 ± 3 mmHg, *p* = 0.027; 39 ± 6 vs. 57 ± 3 %pred., *p* = 0.012, respectively) (Fig. 4). There were no correlations between KL-6 serum levels with age, BMI or GM-CSF autoantibody (data not shown).

### Correlation between MUC1 SNP and disease outcome

**Fig. 5:**
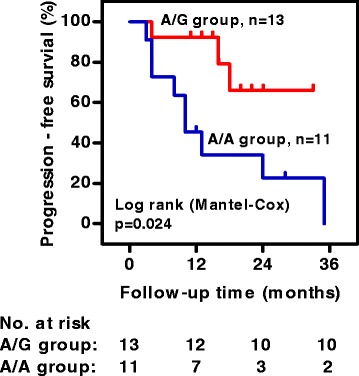
Kaplan-Meier analysis showing disease progression according to MUC1 SNP genotype in 24 PAP patients

PAP patients with A/A genotype had a significantly higher disease progression rate than those with A/G genotype (82 vs. 23 % *p* = 0.006) (Table 2). Kaplan-Meier analysis confirmed the association of MUC1 SNP rs4072037 (A/A) with disease progression in PAP (log-rank test, *p* = 0.024) (Fig. 5).

**Table 2:** Clinical characteristics and disease outcome of PAP patients stratified according to MUC1 rs4072037 gentype

Variables	MUC1 rs4072037 genotype		*p* value
	A/A	A/G	
	*n* = 11	*n* = 13	
Age, years	46 ± 3	46 ± 4	n.s.
Gender, male/female, n	6/5	8/5	n.s.^d^
Smoking history, Current/Non, n	10/1	8/5	n.s.^d^
BMI (kg/m^2^)	25 ± 2	27 ± 1	n.s.
Outcome			
- Disease progression^a^, n (%)	9 (82)	3 (23)	0.006^d^
- Death, n (%)^b^	1 (9)	2 (15)	0.90^d^
- Remission, n (%)	1 (9)	8 (61)	0.07
Treatment, (yes/no)			
- Repeated WLL (>2) during follow-up, n	3	0	0.055^d^
- cumulative number of WLL	7.3 ± 3.1	4.3 ± 1.2	0.359
FVC (% pred)	79 ± 6	82 ± 4	n.s.
FEV_1_ (% pred)	73 ± 4	74 ± 4	n.s.
PaO_2_ (mmHg)	67 ± 6	72 ± 3	n.s.
TLC (% pred)	79 ± 6	83 ± 4	n.s.
SaO_2_ (%)	93 ± 1	94 ± 1	n.s.
A-aDO_2_ (mmHg)	45 ± 4	35 ± 3	0.027
DLco (% pred)	39 ± 6	60 ± 3	0.012
GM-CSF autoantibody (μg/mL)^c^	51 ± 11	53 ± 8	n.s.
KL-6 (U/mL)^c^	8084 ± 1673	2399 ± 462	0.007
LDH (IU/L)^c^	369 ± 38	263 ± 22	0.030

Unless otherwise indicated, values are expressed as mean ± SE

*n.s.* not significant

^a^Disease progression was defined as necessity of whole lung lavage on the basis of deterioration of self-reported symptoms (worsening of dyspnea, cough, chest pain and weight loss) and/or lung function (decrease in FVC or DLco >10 % pred or increase in the A-aDO_2_ > 10 mmHg), and/or chest imaging (increase of the previous findings or appearance of new infiltrates characteristic of PAP) since the last follow-up visit

^b^One death followed whole lung lavage, one was related to alcoholic liver cirrhosis and one to lung cancer

^c^The cut-off of normality for each biomarker is reported in the methods

^d^Fischer’s exact test

### Univariate and multivariate analyses for predicting disease outcome

We performed univariate and multivariate analysis to investigate the association of several factors with disease progression. In the univariate analysis, MUC1 rs4072037 (A/A) was the strongest predictor of disease progression (HR, 4.079; 95 % CI, 1.068–15.571; *p* = 0.040) followed by PaO_2_, A-aDO_2_, DLco, serum KL-6 and LDH (all with HR around 1) (Table 3). In the multivariate analysis, MUC1 rs4072037 (A/A) and gender (male) were significantly associated with disease progression after adjustment for age, gender and A-aDO_2_ as covariates (HR, 5.557, *p* = 0.014 and HR, 5.986, *p* = 0.025), (Table 3).

**Table 3:** Univariate and multivariate Cox proportional hazard model evaluating predictors for disease progression

Variables	β	HR	(95 % CI)	*p* value
Univariate analysis				
rs4072037 (A/A)	1.405	4.079	(1.068–15.571)	0.040
Age (continuous)	−0.001	0.999	(0.944–1.057)	0.966
Gender (male)	1.323	3.755	(0.808–17.444)	0.091
Smoking history (current smoker)	3.480	32.452	(0.101–10395.609)	0.237
BMI (continuous)	−0.056	0.945	(0.837–1.068)	0.367
FVC (continuous)	−0.042	0.959	(0.918–1.001)	0.058
PaO_2_ (continuous)	−0.126	0.882	(0.804–0.967)	0.008
A-aDO_2_ (continuous)	0.086	1.090	(1.015–1.171)	0.017
DLco (continuous)	−0.054	0.947	(0.906–0.997)	0.017
GM-CSF autoantibody (continuous)	−0.023	0.975	(0.938–1.014)	0.201
KL-6 (continuous)	0.001	1.00	(0.99–1.00)	0.014
LDH (continuous)	0.006	1.006	(1.000–1.011)	0.022
Multivariate analysis^a^				
rs4072037 (A/A)	1.715	5.557	(1.412–21.869)	0.014
Gender (male)	1.789	5.986	(1.255–28.557)	0.025

^a^Hazard ratio calculated by the Cox proportional hazard model backward stepwise considering the following variables in the model: age, gender, smoking history (current smoker), baseline FVC (%),baseline DLCO (%), PaO2, KL-6 and LDH

Harrell's C statistic for predicting disease progression significantly increased when MUC1 SNP rs4072037 (A/A) was included into a model with the only remaining covariate gender (male) (C statistic, 0.939; 95 % CI, 0.848–1.030, *p* < 0.001) as compared to gender (male) alone.

## Discussion

The current study showed that the distribution of MUC1 rs4072037 genotypes is different between PAP patients and healthy controls and is correlated with serum KL-6 levels. We also found that the rs4072037 (A/A) genotype is associated with severity of pulmonary dysfunction and disease progression. To our knowledge, this is the first report on a likely association between MUC1 gene SNP, KL-6 levels and disease outcome in Caucasian PAP patients.

It is known that the distribution of rs4072037 genotypes in MUC1 influence serum KL-6 levels and that the distribution of rs4072037 genotype in both healthy subjects and patients with ILD varies with ethnicity, the A/G genotype being more common in Caucasians and the A/A genotype more common in Japanese [20]. In our study, the distribution of the rs4072037 genotypes in healthy controls was different from the distribution in CEU population (Utah residents with ancestry from northern and western Europe populations), which is reported in the HapMap database (International HapMap Project) [31]. In fact, in the CEU population the G/G genotype is less frequent than the A/A genotype (14 vs. 30 %), while in our cohort we found the same frequency for both genotypes (30 %). This distribution is very similar to that reported in the GIH (Gujarati Indians in Houston, Texas) population, which shows a frequency of 26 % for both A/A and G/G genotypes (International HapMap Project) [31].

We did not observe the G/G genotype in our PAP patients. This finding could suggest a correlation between the rs4072037 A allele and susceptibility to PAP, but further exploration is needed.

With regard to the correlation between MUC rs4072037 genotype and serum KL-6 levels, we found that serum KL-6 levels were higher in PAP patients with A/A genotype than in those with A/G genotype, whereas this was not observed in healthy controls. In a previous study with Caucasians (Dutch cohort), KL-6 levels were higher in serum of healthy controls and sarcoidosis patients carrying the G allele, with G homozygotes having the highest levels, A homozygotes the lowest, and heterozygotes intermediate levels, which is compatible with a gene-dose effect [19]. This gene-dose effect was not reported by Horimasu et al. neither in Caucasians nor in Japanese, because serum KL-6 levels varied according to genotype in a non concordant way between healthy subjects and ILD patients [20]. This point needs further investigation.

MUC1 is an extracellular protein anchored to the epithelial surface and involved in morphogenetic signal transduction [32]. Rs4072037 SNP disrupts the physiological functions of MUC1, due to alternative splicing of the 59-region of exon 2 controlled by rs4072037, and ultimately results in failure of the physiological protection of human tissue [33, 34].

The G allele in rs4072037 has been reported to be protective against gastric cancer in the Han Chinese and Caucasian population [35, 36]. The A allele, on the contrary, seems to confer susceptibility to dry eye syndrome and gastric cancer [33, 35–37]. We found that serum KL-6 levels were lower in PAP patients carrying the G allele and that the G allele was associated with better pulmonary function (lower A-aDO_2_ and higher DLco) in PAP patients. In the multivariate analysis, the model including MUC1 rs4072037 A/A genotype showed a significant association with disease progression when added to age, gender, smoking history and A-aDO_2_ as covariates. We can only speculate on the meaning of our findings. Accumulation of surfactant proteins, phospholipids, and cell debris as well as mechanical stress due to impaired alveolar surface tension are underlying mechanisms of epithelial damage in PAP [5]. Reactive hyperplasy of alveolar epithelial cells and altered permeability of air-blood membrane are typical pathologic findings in PAP [38]. Hyperproduction of mucins, like KL-6, or cytokeratins, like CYFRA-21, has been observed in PAP and is considered an injury-repair response to airway epithelial damage [11, 39]. It can be hypothesized that the hyperproduction of aberrant MUC1 isoforms, linked to the presence of rs4072037 (A) homozygosis can lead to failure in epithelial repair and perpetuation of damage in PAP. Apart from this intriguing hypothesis, our results, if confirmed, could suggest a role of MUC1 SNP genotype at least as a genetic biomarker for routine clinical use in PAP.

Limitations of this study are the small sample size of this cohort, and the lack of a validation cohort. Moreover, we cannot exclude that previously received WLL could have an influence on serum KL-6 concentration and therefore on their correlation with MUC1 genotypes.

## Conclusion

On the basis of our results, MUC1 SNP (rs4072037) genotype may be correlated with serum KL-6 levels, the severity of pulmonary dysfunction and disease progression in PAP. Further multicentric studies involving larger populations of different ethnicities are needed to validate these results.

### Ethical standards

The experiments in this study comply with Ethik-Kommission Universitätsklinikum Essen in Germany.

## Abbreviations

A-aDO_2_: Alveolar arterial oxygen gradient
BALF: bronchoalveolar lavage fluid
DLco: diffusing capacity of the lung for carbon monoxide
ELISA: enzyme-linked immunosorbent assay
FEV_1_: forced expiratory volume in one second
FVC: forced vital capacity
GM-CSF: granulocyte macrophage colony stimulating factor
HRCT: high resolution computed tomography
PAP: pulmonary alveolar proteinosis
PFTs: pulmonary function tests
RT-PCR: real-time polymerase chain reaction
SNP: single nucleotide polymorphism
TLC: total lung capacity
WLL: whole lung lavage
